# Benzyl­triethyl­ammonium aqua­trichlorido­zincate

**DOI:** 10.1107/S1600536811048823

**Published:** 2011-11-19

**Authors:** Lei Jin

**Affiliations:** a College of Chemistry and Chemical Engineering, Southeast University, Nanjing 210096, People’s Republic of China

## Abstract

In the crystal structure of the title mol­ecular salt, (C_13_H_22_N)[ZnCl_3_(H_2_O)], the distorted tetrahedral anions are linked by O—H⋯Cl hydrogen bonds, generating [100] chains. Weak cation-to-anion C—H⋯Cl inter­actions generate a three-dimensional network.

## Related literature

For background literature concerning mol­ecular salts, see: Tan *et al.* (2010 ▶); Jin *et al.* (2011 ▶).
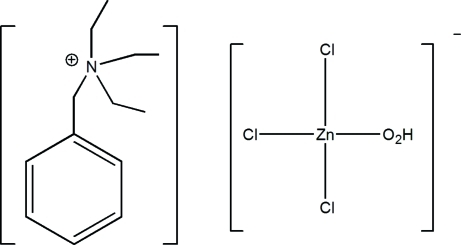


## Experimental

### 

#### Crystal data

(C_13_H_22_N)[ZnCl_3_(H_2_O)]
*M* *_r_* = 382.05
Orthorhombic, 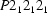

*a* = 8.3236 (17) Å
*b* = 13.484 (3) Å
*c* = 15.808 (3) Å
*V* = 1774.2 (6) Å^3^
*Z* = 4
Mo *K*α radiation
μ = 1.83 mm^−1^
*T* = 291 K
0.28 × 0.24 × 0.22 mm

#### Data collection

Rigaku Mercury2 CCD diffractometer
Absorption correction: multi-scan (*CrystalClear*; Rigaku, 2005 ▶) *T*_min_ = 0.629, *T*_max_ = 0.689
18427 measured reflections
4054 independent reflections
3522 reflections with *I* > 2σ(*I*)
*R*_int_ = 0.045

#### Refinement

*R*[*F* ^2^ > 2σ(*F* ^2^)] = 0.034
*wR*(*F* ^2^) = 0.077
*S* = 1.09
4054 reflections
177 parameters
H-atom parameters constrained
Δρ_max_ = 0.27 e Å^−3^
Δρ_min_ = −0.37 e Å^−3^
Absolute structure: Flack (1983 ▶), 1735 Friedel pairs
Flack parameter: 0.022 (13)

### 

Data collection: *CrystalClear* (Rigaku, 2005 ▶); cell refinement: *CrystalClear*; data reduction: *CrystalClear*; program(s) used to solve structure: *SHELXS97* (Sheldrick, 2008 ▶); program(s) used to refine structure: *SHELXL97* (Sheldrick, 2008 ▶); molecular graphics: *SHELXTL* (Sheldrick, 2008 ▶); software used to prepare material for publication: *SHELXTL*.

## Supplementary Material

Crystal structure: contains datablock(s) I, global. DOI: 10.1107/S1600536811048823/hb6492sup1.cif

Structure factors: contains datablock(s) I. DOI: 10.1107/S1600536811048823/hb6492Isup2.hkl

Additional supplementary materials:  crystallographic information; 3D view; checkCIF report

## Figures and Tables

**Table 1:** Hydrogen-bond geometry (Å, °)

*D*—H⋯*A*	*D*—H	H⋯*A*	*D*⋯*A*	*D*—H⋯*A*
O1—H1*D*⋯Cl2^i^	0.98	2.17	3.121 (2)	163
O1—H1*E*⋯Cl1^ii^	0.93	2.24	3.155 (2)	168
C1—H1*B*⋯Cl1^iii^	0.96	2.82	3.599 (3)	139

Symmetry codes: (i) 
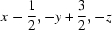
; (ii) 
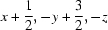
; (iii) 
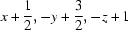
.

**Fig. 1.**
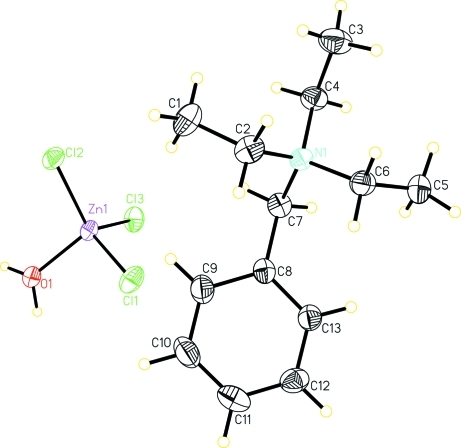
The molecular structure of (I) showing 30% probability displacement ellipsoids.

## supplementary crystallographic information

### Experimental

In room temperature benzyltriethylammoniumchlorine (10 mmol, 2.28 g) were dissolved in 30 ml water, then a solution with ZnCl_2_(5 mmol, 0.68 g) was dropped slowly into the previous solution with properly sirring. Single crystals suitable for X-ray structure analysis were obtained by the slow evaporation of the above solution after two weeks in air with some colorless solid blocks appeared after days with yield about 75%.

The dielectric constant of the compound as a function of temperature indicates that the permittivity is basically temperature-independent (ε = C/(T–T_0_)), suggesting that this compound is not ferroelectric or there may be no distinct phase transition occurring within the measured temperature (below the melting point).

### Refinement

H atoms were placed in calculated positions(C—H = 0.93Å for C*sp^2^* atoms and C—H = 0.96Å and 0.97 Å for C*sp^3^* atoms), assigned fixed *U*_iso_ values [*U*_iso_ = 1.2*U*eq(C*sp^2^*/N) and 1.5*U*eq(C*sp^3^*)] and allowed to ride.

### Crystal data

**Table:**

(C_13_H_22_N)[ZnCl_3_(H_2_O)]	*F*(000) = 792
*M**_r_* = 382.05	*D*_x_ = 1.430 Mg m^−^^3^
Orthorhombic, *P*2_1_2_1_2_1_	Mo *K*α radiation, λ = 0.71073 Å
Hall symbol: P 2ac 2ab	θ = 3.0–27.5°
*a* = 8.3236 (17) Å	µ = 1.83 mm^−^^1^
*b* = 13.484 (3) Å	*T* = 291 K
*c* = 15.808 (3) Å	Block, colorless
*V* = 1774.2 (6) Å^3^	0.28 × 0.24 × 0.22 mm
*Z* = 4	

### Data collection

**Table:**

Rigaku Mercury2 CCD diffractometer	4054 independent reflections
Radiation source: fine-focus sealed tube	3522 reflections with *I* > 2σ(*I*)
graphite	*R*_int_ = 0.045
Detector resolution: 13.6612 pixels mm^-1^	θ_max_ = 27.5°, θ_min_ = 3.0°
CCD_Profile_fitting scans	*h* = −10→10
Absorption correction: multi-scan (*CrystalClear*; Rigaku, 2005)	*k* = −17→17
*T*_min_ = 0.629, *T*_max_ = 0.689	*l* = −20→20
18427 measured reflections	

### Refinement

**Table:**

Refinement on *F*^2^	Secondary atom site location: difference Fourier map
Least-squares matrix: full	Hydrogen site location: inferred from neighbouring sites
*R*[*F*^2^ > 2σ(*F*^2^)] = 0.034	H-atom parameters constrained
*wR*(*F*^2^) = 0.077	*w* = 1/[σ^2^(*F*_o_^2^) + (0.0349*P*)^2^] where *P* = (*F*_o_^2^ + 2*F*_c_^2^)/3
*S* = 1.09	(Δ/σ)_max_ < 0.001
4054 reflections	Δρ_max_ = 0.27 e Å^−^^3^
177 parameters	Δρ_min_ = −0.37 e Å^−^^3^
0 restraints	Absolute structure: Flack (1983), 1735 Friedel pairs
Primary atom site location: structure-invariant direct methods	Flack parameter: 0.022 (13)

### Special details

**Table:**

Geometry. All e.s.d.'s (except the e.s.d. in the dihedral angle between two l.s. planes) are estimated using the full covariance matrix. The cell e.s.d.'s are taken into account individually in the estimation of e.s.d.'s in distances, angles and torsion angles; correlations between e.s.d.'s in cell parameters are only used when they are defined by crystal symmetry. An approximate (isotropic) treatment of cell e.s.d.'s is used for estimating e.s.d.'s involving l.s. planes.
Refinement. Refinement of *F*^2^ against ALL reflections. The weighted *R*-factor *wR* and goodness of fit *S* are based on *F*^2^, conventional *R*-factors *R* are based on *F*, with *F* set to zero for negative *F*^2^. The threshold expression of *F*^2^ > σ(*F*^2^) is used only for calculating *R*-factors(gt) *etc*. and is not relevant to the choice of reflections for refinement. *R*-factors based on *F*^2^ are statistically about twice as large as those based on *F*, and *R*- factors based on ALL data will be even larger.

### Fractional atomic coordinates and isotropic or equivalent isotropic displacement parameters (Å^2^)

**Table:**

	*x*	*y*	*z*	*U*_iso_*/*U*_eq_	
C1	1.0907 (4)	0.6986 (3)	0.6677 (2)	0.0704 (9)	
H1A	1.1371	0.6335	0.6665	0.106*	
H1B	1.1646	0.7440	0.6937	0.106*	
H1C	1.0684	0.7200	0.6110	0.106*	
C2	0.9359 (4)	0.6962 (2)	0.71813 (18)	0.0526 (7)	
H2A	0.9607	0.6758	0.7755	0.063*	
H2B	0.8929	0.7630	0.7208	0.063*	
C3	0.9403 (5)	0.4758 (2)	0.7474 (2)	0.0691 (10)	
H3A	1.0262	0.5159	0.7691	0.104*	
H3B	0.9807	0.4112	0.7333	0.104*	
H3C	0.8578	0.4696	0.7896	0.104*	
C4	0.8714 (3)	0.52380 (19)	0.66941 (17)	0.0485 (7)	
H4A	0.7847	0.4827	0.6482	0.058*	
H4B	0.9541	0.5262	0.6262	0.058*	
C5	0.5348 (4)	0.5569 (2)	0.7260 (2)	0.0631 (8)	
H5A	0.5022	0.5662	0.6683	0.095*	
H5B	0.4453	0.5694	0.7628	0.095*	
H5C	0.5714	0.4900	0.7338	0.095*	
C6	0.6706 (3)	0.6286 (2)	0.74693 (16)	0.0482 (6)	
H6A	0.6267	0.6951	0.7503	0.058*	
H6B	0.7131	0.6119	0.8023	0.058*	
C7	0.7509 (3)	0.66402 (18)	0.59743 (15)	0.0425 (6)	
H7A	0.8416	0.6621	0.5589	0.051*	
H7B	0.6717	0.6173	0.5765	0.051*	
C8	0.6785 (3)	0.76674 (19)	0.59399 (15)	0.0412 (6)	
C9	0.7711 (4)	0.8488 (2)	0.57179 (18)	0.0594 (8)	
H9	0.8812	0.8415	0.5639	0.071*	
C10	0.7015 (5)	0.9410 (2)	0.5613 (2)	0.0720 (10)	
H10	0.7649	0.9950	0.5462	0.086*	
C11	0.5380 (5)	0.9536 (2)	0.57303 (18)	0.0646 (9)	
H11	0.4911	1.0156	0.5657	0.078*	
C12	0.4475 (4)	0.8747 (2)	0.59527 (18)	0.0566 (8)	
H12	0.3378	0.8831	0.6040	0.068*	
C13	0.5148 (4)	0.7814 (2)	0.60535 (16)	0.0481 (6)	
H13	0.4496	0.7281	0.6199	0.058*	
Cl1	0.82104 (9)	0.72961 (7)	0.14959 (5)	0.0678 (2)	
Cl2	1.23689 (9)	0.63181 (6)	0.11597 (5)	0.0603 (2)	
Cl3	0.89340 (10)	0.55114 (6)	−0.02493 (5)	0.0620 (2)	
N1	0.8077 (2)	0.62856 (15)	0.68371 (12)	0.0372 (5)	
O1	1.0364 (2)	0.78841 (15)	−0.01861 (15)	0.0646 (6)	
H1D	0.9476	0.8264	−0.0434	0.136 (18)*	
H1E	1.1189	0.7929	−0.0579	0.120 (16)*	
Zn1	1.00040 (4)	0.66907 (2)	0.056683 (18)	0.04478 (10)	

### Atomic displacement parameters (Å^2^)

**Table:**

	*U*^11^	*U*^22^	*U*^33^	*U*^12^	*U*^13^	*U*^23^
C1	0.0440 (17)	0.089 (2)	0.078 (2)	−0.0088 (17)	−0.0040 (16)	−0.007 (2)
C2	0.0519 (16)	0.0584 (17)	0.0473 (15)	−0.0004 (14)	−0.0111 (13)	−0.0131 (14)
C3	0.083 (2)	0.061 (2)	0.0629 (19)	0.0251 (17)	0.0060 (17)	0.0056 (16)
C4	0.0562 (18)	0.0413 (15)	0.0479 (15)	0.0091 (13)	0.0042 (13)	−0.0071 (12)
C5	0.055 (2)	0.0617 (17)	0.0730 (19)	−0.0036 (15)	0.0157 (15)	0.0108 (16)
C6	0.0527 (16)	0.0529 (15)	0.0389 (14)	0.0097 (14)	0.0095 (12)	0.0028 (12)
C7	0.0488 (14)	0.0457 (13)	0.0331 (12)	−0.0015 (13)	−0.0011 (11)	−0.0044 (11)
C8	0.0447 (14)	0.0463 (14)	0.0326 (12)	−0.0064 (12)	−0.0026 (11)	0.0016 (11)
C9	0.0538 (18)	0.0612 (18)	0.0630 (19)	−0.0098 (15)	0.0029 (15)	0.0122 (15)
C10	0.082 (3)	0.0537 (18)	0.080 (2)	−0.0145 (18)	−0.002 (2)	0.0219 (18)
C11	0.091 (3)	0.0474 (16)	0.0556 (17)	0.0096 (17)	−0.0104 (17)	0.0049 (14)
C12	0.0546 (18)	0.0683 (19)	0.0469 (16)	0.0142 (15)	−0.0009 (13)	0.0061 (15)
C13	0.0450 (16)	0.0538 (15)	0.0456 (14)	−0.0046 (15)	−0.0037 (14)	0.0050 (11)
Cl1	0.0499 (4)	0.0890 (6)	0.0646 (5)	−0.0015 (4)	0.0090 (4)	−0.0227 (4)
Cl2	0.0412 (4)	0.0740 (5)	0.0658 (5)	−0.0034 (4)	−0.0119 (3)	0.0041 (4)
Cl3	0.0608 (5)	0.0689 (5)	0.0565 (4)	−0.0192 (4)	−0.0050 (4)	−0.0113 (4)
N1	0.0378 (11)	0.0402 (10)	0.0337 (10)	0.0039 (10)	0.0030 (9)	−0.0056 (9)
O1	0.0423 (12)	0.0623 (12)	0.0893 (15)	−0.0024 (10)	0.0078 (11)	0.0192 (12)
Zn1	0.03494 (16)	0.05128 (17)	0.04811 (17)	−0.00472 (16)	−0.00111 (15)	−0.00287 (12)

### Geometric parameters (Å, °)

**Table:**

C1—C2	1.515 (4)	C7—C8	1.511 (4)
C1—H1A	0.9600	C7—N1	1.521 (3)
C1—H1B	0.9600	C7—H7A	0.9700
C1—H1C	0.9600	C7—H7B	0.9700
C2—N1	1.506 (3)	C8—C13	1.389 (4)
C2—H2A	0.9700	C8—C9	1.394 (4)
C2—H2B	0.9700	C9—C10	1.382 (4)
C3—C4	1.506 (4)	C9—H9	0.9300
C3—H3A	0.9600	C10—C11	1.384 (5)
C3—H3B	0.9600	C10—H10	0.9300
C3—H3C	0.9600	C11—C12	1.350 (5)
C4—N1	1.526 (3)	C11—H11	0.9300
C4—H4A	0.9700	C12—C13	1.386 (4)
C4—H4B	0.9700	C12—H12	0.9300
C5—C6	1.523 (4)	C13—H13	0.9300
C5—H5A	0.9600	Cl1—Zn1	2.2478 (8)
C5—H5B	0.9600	Cl2—Zn1	2.2373 (9)
C5—H5C	0.9600	Cl3—Zn1	2.2330 (8)
C6—N1	1.517 (3)	O1—Zn1	2.024 (2)
C6—H6A	0.9700	O1—H1D	0.9808
C6—H6B	0.9700	O1—H1E	0.9280
			
C2—C1—H1A	109.5	N1—C7—H7A	108.2
C2—C1—H1B	109.5	C8—C7—H7B	108.2
H1A—C1—H1B	109.5	N1—C7—H7B	108.2
C2—C1—H1C	109.5	H7A—C7—H7B	107.3
H1A—C1—H1C	109.5	C13—C8—C9	117.5 (3)
H1B—C1—H1C	109.5	C13—C8—C7	121.1 (2)
N1—C2—C1	115.2 (2)	C9—C8—C7	121.1 (2)
N1—C2—H2A	108.5	C10—C9—C8	120.9 (3)
C1—C2—H2A	108.5	C10—C9—H9	119.6
N1—C2—H2B	108.5	C8—C9—H9	119.6
C1—C2—H2B	108.5	C9—C10—C11	120.4 (3)
H2A—C2—H2B	107.5	C9—C10—H10	119.8
C4—C3—H3A	109.5	C11—C10—H10	119.8
C4—C3—H3B	109.5	C12—C11—C10	119.2 (3)
H3A—C3—H3B	109.5	C12—C11—H11	120.4
C4—C3—H3C	109.5	C10—C11—H11	120.4
H3A—C3—H3C	109.5	C11—C12—C13	121.3 (3)
H3B—C3—H3C	109.5	C11—C12—H12	119.4
C3—C4—N1	114.1 (2)	C13—C12—H12	119.4
C3—C4—H4A	108.7	C12—C13—C8	120.7 (3)
N1—C4—H4A	108.7	C12—C13—H13	119.6
C3—C4—H4B	108.7	C8—C13—H13	119.6
N1—C4—H4B	108.7	C2—N1—C6	107.17 (19)
H4A—C4—H4B	107.6	C2—N1—C7	110.7 (2)
C6—C5—H5A	109.5	C6—N1—C7	110.93 (19)
C6—C5—H5B	109.5	C2—N1—C4	111.6 (2)
H5A—C5—H5B	109.5	C6—N1—C4	111.1 (2)
C6—C5—H5C	109.5	C7—N1—C4	105.44 (18)
H5A—C5—H5C	109.5	Zn1—O1—H1D	122.6
H5B—C5—H5C	109.5	Zn1—O1—H1E	123.8
N1—C6—C5	114.5 (2)	H1D—O1—H1E	104.8
N1—C6—H6A	108.6	O1—Zn1—Cl3	106.59 (7)
C5—C6—H6A	108.6	O1—Zn1—Cl2	107.13 (6)
N1—C6—H6B	108.6	Cl3—Zn1—Cl2	115.66 (4)
C5—C6—H6B	108.6	O1—Zn1—Cl1	101.18 (7)
H6A—C6—H6B	107.6	Cl3—Zn1—Cl1	111.79 (3)
C8—C7—N1	116.38 (19)	Cl2—Zn1—Cl1	113.09 (3)
C8—C7—H7A	108.2		
			
N1—C7—C8—C13	−90.3 (3)	C1—C2—N1—C7	−64.5 (3)
N1—C7—C8—C9	95.6 (3)	C1—C2—N1—C4	52.7 (3)
C13—C8—C9—C10	−0.3 (4)	C5—C6—N1—C2	−175.1 (2)
C7—C8—C9—C10	174.1 (3)	C5—C6—N1—C7	63.9 (3)
C8—C9—C10—C11	0.3 (5)	C5—C6—N1—C4	−53.0 (3)
C9—C10—C11—C12	0.3 (5)	C8—C7—N1—C2	−60.2 (3)
C10—C11—C12—C13	−0.9 (5)	C8—C7—N1—C6	58.7 (3)
C11—C12—C13—C8	0.9 (4)	C8—C7—N1—C4	179.0 (2)
C9—C8—C13—C12	−0.3 (4)	C3—C4—N1—C2	57.5 (3)
C7—C8—C13—C12	−174.7 (2)	C3—C4—N1—C6	−62.0 (3)
C1—C2—N1—C6	174.4 (2)	C3—C4—N1—C7	177.8 (3)

### Hydrogen-bond geometry (Å, °)

**Table:**

*D*—H···*A*	*D*—H	H···*A*	*D*···*A*	*D*—H···*A*
O1—H1D···Cl2^i^	0.98	2.17	3.121 (2)	163
O1—H1E···Cl1^ii^	0.93	2.24	3.155 (2)	168
C1—H1B···Cl1^iii^	0.96	2.82	3.599 (3)	139

Symmetry codes: (i) *x*−1/2, −*y*+3/2, −*z*; (ii) *x*+1/2, −*y*+3/2, −*z*; (iii) *x*+1/2, −*y*+3/2, −*z*+1.
